# 2235: Equipping health professional students to apply pharmacogenomic data to clinical decision making in real-world scenarios: Comparison of an active engagement Versus didactic teaching approach

**DOI:** 10.1017/cts.2017.166

**Published:** 2018-05-10

**Authors:** Meghan J. Arwood, Caitrin Rowe McDonough, Larisa H. Cavallari, Amanda R. Elsey, Reginald F. Frye, Yan Gong, Julie A. Johnson, Kristin W. Weitzel, Taimour Langaee

**Affiliations:** University of Florida Clinical and Translational Science Institute, Gainesville, FL, USA

## Abstract

OBJECTIVES/SPECIFIC AIMS: Compare effectiveness of a patient case-based, interactive teaching approach that included optional student genotyping with traditional didactic teaching strategies for increasing students’ knowledge and ability to effectively use pharmacogenomic data in clinical decision making. METHODS/STUDY POPULATION: The UF College of Pharmacy offers a required Personalized Medicine (PM) course for pharmacy students as well as an elective course, Clinical Applications of Personalized Medicine (CAPM). Students dual enrolled in the PM and elective CAPM courses comprised the intervention (INT) group, with interactive patient case-based teaching and the option to undergo personal genotyping, whereas students enrolled in PM alone comprised the control (CTR) group, which primarily used a traditional didactic teaching format and did not include personal genotyping. Both groups completed a pre- and post-course patient case-based test (15 questions/1 point each) to evaluate their knowledge and abilities to apply genotype and other patient-specific data to drug therapy recommendations. Pre- and post-course test scores for knowledge were compared between the INT and CTR groups using the Student *t*-test. RESULTS/ANTICIPATED RESULTS: In total, 52 students completed surveys (INT group, n=21; CTR group, n=31). Race was similar between groups, but there were fewer females in the INT compared with CTR group (8 vs. 22, *p*=0.02). Pre-course knowledge scores did not differ between INT and CTR groups (6.8±2.2 vs. 6.3±1.6 respectively, *p*=0.34), however, post-course scores were significantly higher in the INT Versus CTR group (10.0±2.3 vs. 7.5±1.7, *p*<0.0001). DISCUSSION/SIGNIFICANCE OF IMPACT: There have been significant advancements in the clinical applications of pharmacogenomic and genomic data, however, barriers to routine clinical adoption of genomic medicine persist. Developing education and training methods that equip practitioners to effectively translate genomic data into evidence-based clinical recommendations has been identified as a key strategy to overcome such barriers. Our data suggest that a personalized medicine course that employs patient-centered, case-based teaching strategies and includes optional personal genotyping for students compared with traditional didactic instruction improves students’ knowledge and abilities to apply pharmacogenomic data in practice-based scenarios. These results can inform future strategies for educating healthcare professionals on the clinical use of pharmacogenomic and genomic data.

